# ‘Don't play the butter notes’: jazz in medical education

**DOI:** 10.3402/meo.v21.30582

**Published:** 2016-04-18

**Authors:** Melissa Bradner, Darryl V. Harper, Mark H. Ryan, Allison A. Vanderbilt

**Affiliations:** 1 Department of Family Medicine and Population Health, Virginia Commonwealth University, Richmond, VA, USA; 2 Department of Music, Virginia Commonwealth University, Richmond, VA, USA; 3 Department of Family Medicine, College of Medicine and Life Sciences, University of Toledo, Toledo, OH, USA

**Keywords:** medical education, clinical education, communication skills, mentoring

## Abstract

Jazz has influenced world music and culture globally – attesting to its universal truths of surviving, enduring, and triumphing over tragedy. This begs the question, what can we glean in medical education from this philosophy of jazz mentoring? Despite our training to understand disease and illness in branching logic diagrams, the human experience of illness is still best understood when told as a story. Stories like music have tempos, pauses, and silences. Often they are not linear but wrap around the past, future, and back to the present, frustrating the novice and the experienced clinician in documenting the history of present illness. The first mentoring lesson Hancock discusses is from a time he felt stuck with his playing – his sound was routine. Miles Davis told him in a low husky murmur, ‘Don't play the butter notes’. In medical education, ‘don't play the butter notes’ suggests not undervaluing the metacognition and reflective aspects of medical training that need to be fostered during the early years of clinical teaching years.

In 2014, jazz master Herbie Hancock delivered the Harvard Norton Lecture series called, ‘The Ethics of Jazz’ (1). In this series of talks, Hancock discusses the mentorship he received from Miles Davis in the early years of his professional life as a jazz pianist. Jazz has influenced world music and culture globally – attesting to its universal truths of surviving, enduring, and triumphing over tragedy. This begs the question, what can we glean in medical education from this philosophy of jazz mentoring?

Despite our training to understand disease and illness in branching logic diagrams, the human experience of illness is still best understood when told as a story. Stories like music have tempos, pauses, and silences. Often they are not linear but wrap around the past, future, and back to the present, frustrating the novice and the experienced clinician in documenting the history of present illness. In his essay ‘Exploding the Narrative’, another iconic jazz pianist, Vijay Iyer, writes, ‘Narrativity is in the moment-to-moment act of making the music. The act of musical balance in the face of arduous challenges tells a compelling, even richly symbolic story. For what one hears is necessarily the result of much effort, time, and process – of *labor*’ (2).

Similarly, Ralph Stacy's *Complex Responsive Processes of Relating Theory* describes the back and forth of patient and physician dialog as: ‘mutual expectations of associative response; turn-taking sequences; sequencing, segmenting and categorizing actions; rhetorical devices’ (3). The patient story is a creation, like a musical composition, that is unique to that moment and the participants.

The first mentoring lesson Hancock discusses is from a time he felt stuck with his playing – his sound was routine. Miles Davis told him in a low husky murmur, ‘Don't play the butter notes’. Hancock pondered these words for a long time trying to understand their significance. He finally came to interpret this advice as the easy, expected, or routine notes. Miles Davis was telling him to look beyond the obvious notes on the musical score and stretch himself to find a deeper, more meaningful expression of the music that was hidden in the text.

In medical education, ‘don't play the butter notes’ suggests not undervaluing the metacognition and reflective aspects of medical training that need to be fostered during the early years of clinical teaching years (4). The ‘butter notes’ are the differential diagnoses workup of a patient's chief complaint – core knowledge that every medical student needs to master for multiple-choice tests and national board requirements. The artful medical student needs to be coached to find the more subtle notes present in any patient encounter. Unlike the differential diagnosis, each patient is unique, the illness is unique, the story is unique – these themes run as an undercurrent to any patient presentation. For example, an older male patient seen with a student comes in for benign prostatic hypertrophy, hypertension, and depression, that is, the butter notes. These issues are addressed, but what the patient wants to talk about is his anguish at putting his 90-year-old mother in a nursing home; how she fell at the first nursing home that was providing poor care, and was hospitalized for a week; how he found a better place for her and is now pleased with her care. These points of the patient's story are like the alternate chords Herbie Hancock was playing when he dropped ‘the butter notes’ and what makes the day in and day out of medical care interesting. Learning to look beyond the superficial or obvious elements of a medical visit (such as high blood pressure, headaches, or BPH) allows clinicians to detect underlying issues that are key to a given medical visit.

In her book *A Case for God*, Karen Armstrong argues:

Scientific rationalism consists largely of problem solving, an approach that does lead to systematic advance … But the humanities do not function in this way, because the problems they confront, such as mortality, grief, evil, or the nature of happiness, are not capable of a once-and-for-all solution … The French philosopher Gabriel Marcel (1889–1973) distinguished between a *problem*, “something met which bars my passage” and “is before me in its entirety,” and a *mystery*, “something in which I find myself caught up, and whose essence is not before me in its entirety.” … It is always possible – and perhaps a modern temptation – to turn a mystery into a problem and try to solve it by applying the appropriate technique. (5)

The butter notes address the problem.

Armstrong then writes, ‘… Like religion, at its best, music marks the “limits of reason”… Music goes beyond the reach of words: it is not about anything’. In other words, music, at its best, addresses the mystery.

Another jazz pearl Mr. Hancock discusses is, ‘I always listen to what I can leave out; the loudest noise in the world is silence’. The greatest connection you can make with a patient is profound, deep listening – the art of silence. Silence and listening allow the patient room to share the undercurrents of the encounter. Illness is often described as a disruption of the patient's self-narrative; and by allowing time and space for the patient to tell a story, the physician nurtures wholeness. As described in the book, *Narrative in Health Care*, the patient ‘approaches the caregiver with hope for redress in order that the state of affairs in her life might be restored, and that the plot of her life story might be reconstituted’ (6). During clinical teaching, a preceptor should comment on the power of silence, ask the student to look for the impact of silence.

‘Don't judge the unexpected:’ Mr. Hancock tells a story of when he felt the band was on a great roll in a particular song – they were all improvising – then it was his turn to take the lead and he hit the wrong note – not just the wrong note – a terrible note. But what happened next amazed him – Miles Davis didn't judge his mistake; rather he ran with it and took the music in a new direction. In medical education, we could think of this precept as a way we could address medical errors. Medical education has not effectively grappled with training students on how to address medical errors. The hidden medical culture of discomfort and lack of disclosure of errors in medicine has been well documented (7). What if we trained students like Miles Davis and considered errors as an opportunity for greater learning, as a continuation of dialog, as a way to probe a student's understanding of why he or she made the incorrect answer, to see if an error could lead to a new understanding, maybe a research proposal?

In conclusion, why did Mr. Hancock title his talk, the ‘ethics’ of jazz? ‘Ethics’ implies a moral code, a set of values and standards. Does music have morals? We suggest that the ethics he describes relate to how the music is created, how jazz is passed from one generation to the next, that is, the ethics of mentorship. Mr. Hancock chooses this title because the human behaviors he describes are central to the music – they are the music – inseparable. You would think in medicine the ideas we have been discussing here would also be central to the education and practice of medicine, and how it is passed from one generation of physicians to the next. In the world of biomedical medicine, however, the relational aspects of medicine have taken a back seat for many years. In the words of a Nobel laureate physician, Bernard Lown:

… the doctor, by virtue of accepting science so totally, creates a total imbalance, forgetting the art of healing, forgetting the art of engagement, forgetting the art of listening, forgetting the art of caring and ceasing to invest time with the patient. So I believe that medicine has lost its human face. (8)

Medical school curricula make an effort to incorporate more relational aspects of medicine but they are shadowed by the overwhelming amounts of biomedical information students need to master. Students learn early to spend more time and effort on high-stakes courses with multiple-choice exams. How appropriate then that jazz might coax physicians to think about the art of listening, how it creates stories in time, and what stories (or music) have to do with human healing?
